# Computational evaluation of efflux pump homologues and lignans as potent inhibitors against multidrug-resistant *Salmonella typhi*

**DOI:** 10.1371/journal.pone.0303285

**Published:** 2024-06-25

**Authors:** Iqra Shafique, Mehak Rafiq, Nosheen Fatima Rana, Farid Menaa, Fatemah Almalki, Alya Aljuaid, Sulaiman Mohammed Alnasser, Amenah S. Alotaibi, Madahiah Bint E. Masood, Tahreem Tanweer

**Affiliations:** 1 Department of Biomedical Engineering and Sciences, School of Mechanical & Manufacturing Engineering, National University of Science & Technology, Islamabad, Pakistan; 2 School of Interdisciplinary Engineering & Sciences (SINES), National University of Science & Technology, Islamabad, Pakistan; 3 Department of Medicine and Nanomedicine, California Innovations Corporation, San Diego, CA, United States of America; 4 Department of Biology, College of Science and Humanities, Shaqra University, Al Quwaiiyah, Saudi Arabia; 5 Department of Pharmacology, Unaizah College of Pharmacy, Qassim University, Buraydah, Saudi Arabia; 6 Department of Biology, Genomic & Biotechnology Unit, Faculty of Science, University of Tabuk, Tabuk, Saudi Arabia; Ahram Canadian University, EGYPT

## Abstract

Typhoid fever, caused by *Salmonella* enterica serovar *typhi*, presents a substantial global health threat, particularly in regions with limited healthcare infrastructure. The rise of multidrug-resistant strains of *S. typhi* exacerbates this challenge, severely compromising conventional treatment efficacy due to over activity of efflux pumps. In our study, a comprehensive exploration of two fundamental aspects to combat MDR in *S. typhi* is carried out; i.e. employing advanced bioinformatics analyses and AlphaFold AI, We successfully identified and characterised a putative homologue, ABC-TPA, reminiscent of the P-glycoprotein (P-gp) known for its role in multidrug resistance in diverse pathogens. This discovery provides a critical foundation for understanding the potential mechanisms driving antibiotic resistance in *S. typhi*. Furthermore, employing computational methodologies, We meticulously assessed the potential of lignans, specifically Schisandrin A, B, and C, as promising Efflux Pump Inhibitors (EPIs) against the identified P-gp homologue in *S. typhi*. Noteworthy findings revealed robust binding interactions of Schisandrin A and B with the target protein, indicating substantial inhibitory capabilities. In contrast, Schisandrin C exhibited instability, showing varied effectiveness among the evaluated lignans. Pharmacokinetics and toxicity predictions underscored the favourable attributes of Schisandrin A, including prolonged action duration. Furthermore, high systemic stability and demanished toxicity profile of SA and SB present their therapeutic efficacy against MDR. This comprehensive investigation not only elucidates potential therapeutic strategies against MDR strains of *S. typhi* but also highlights the relevance of computational approaches in identifying and evaluating promising candidates. These findings lay a robust foundation for future empirical studies to address the formidable challenges antibiotic resistance poses in this clinically significant infectious diseases.

## Introduction

*Salmonella enterica serotype typhi* is a gram-negative bacterium and multi-drug resistance strain that causes enteric or typhoid fever [1]. As a result, 16–20 million cases (600,000 fatalities) of typhoid burden occur every year globally, primarily in Asian countries and some regions of Africa, due to poor hygiene standards as it’s transmission through faecal-oral route [2–5].

Antibiotic therapy is the gold standard of treatment; nevertheless, overusing them can produce a variety of side effects as well as the selection of resistant microorganisms [6]. Furthermore, antibiotic resistance is a natural phenomenon in the microorganism, induced by mutations or horizontal transfer of genetic material, demonstrating the correlation between drug use and pathogenic resistance [7, 8]. The mechanism behind the MDR is the efflux of drugs from cells through the ATP binding cassette (ABC) transporters, which act as integral membrane pumps [9]. They are formed by combining numerous transmembrane proteins engaged in active molecule pumping [7, 10]. Therefore, they play an essential role in the evolution of MDR in bacteria [11]. Bacterial drug efflux pumps are an issue in treating bacterial infections because they can transport drugs or other substrates outside the cells, lowering the drug concentrations [12].

Five superfamilies of multi-drug efflux pumps in prokaryotes can play role in MDR [13]. However, most multi-drug efflux pumps in eukaryotes belong to the ATP-binding cassette (ABC) family of transporters. The (ABC) transporters are the most important class of proteins in plasma membrane organelles of all living cells and occasionally in gram-negative bacteria [7]. These ubiquitous membrane proteins utilize the energy produced from the hydrolysis of ATP and mediate the export of ions, sugars, amino acids, drugs, polysaccharides, and proteins [14–16]. The most typical ABC transporter is P-glycoprotein (P-gp; 170 kDa), coded by the ABCB-1 (MDR-1) gene [17]. It affects the pharmacokinetics of numerous drugs and is implicated in MDR of many human cancers, HIV, and epileptic diseases [18, 19]. Orthologously similar proteins to P-gp have been reported in many different organisms, such as, LmrA in *Lactobacillus Lactics*, MsbA in *Escherichia* *coli* and Sav1866 in *Staphylococcus aureus* have been reported [20, 21].

Phytocomponents may have minimal antimicrobial activities, but they may reinforce the antimicrobial potential of other antibiotics. Such combination therapies are exclusively being explored as MDR in *S.typhi* is reported to elevate risk of death to 70%. Thus, phytocompound-based Efflux pump inhibitors (EPIs) must be understood to prevent drug efflux, revive their activity, and enhance lethality at low dosages [22, 23].

Several research groups have described the ability of natural products, such as lignans and, flavonoids, alkaloids, to inhibit ABC transporters P-gp mediated efflux and restore drug sensitivity in MDR cells [11]. Extracts such as *Schisandra chinensis* are rich in lignans have various biological activities, including antioxidant, anti-inflammatory and anticancer activities. Three types of Schisandrin are reported such as Schisandrin A, Schisandrin B and Schisandrin C. Schisandrin, a naturally occurring lignan from *Schisandra chinensis*, substantially inhibits ABC transporter proteins in cancer cell lines. The primary antibacterial components of the *Schisandra chinensis* are lignans, namely, SA possesses potent anti-inflammatory, antioxidant, and anticancer activities and exhibits a variety of other pharmacological effects, such as neuroprotection, hepatoprotection, and antidiabetic. SB can reverse drug resistance by inhibiting P-gp. SC has anti-inflammatory effects and inhibits NFAT transcription [24, 25]. Although *S. chinensis* extract have been reported to have antimicrobial effects against *E. coli, B. subtilis, Salmonella and S. aureus*, and *ciprofloxacin-resistant E.coli* [26, 27].

The present study aimed to evaluate the effectiveness of lignans SA, SB, and SC as EPI of P-gp homologue in *S. typhi*. We identified TPA as a homologue of P-gp in *S. typhi* and investigated the binding affinity of SA, SB, and SC. We report a comprehensive analysis: Identification of target protein, prediction of three-dimensional structure of *S. typhi* ABC transporter TPA protein by molecular modelling and its refinement with Molecular dynamic (MD) simulation, Docking with Schisandrins analogues and MD simulation of Docked complexes at 100 ns are carried out.

## Methodology

### Bioinformatics tools

Open Babel GUI, UCSF Chimera 1.8.1, Pubchem (www.pubchem.com (accessed on May 15 2021), Clustal omega, RCSB PDB (http://www.rscb.org/pdb (accessed on May 15 2021), SAVES v6.0, CB dock, Alpha Fold and Discovery Studio, Gromacs 5.1 were used in the present investigation.

#### Protein identification and modeling

*Sequence retrieval and template selection*. Since the *S. typhi* ABC transporter protein’s experimental 3D structure is unknown, modelling techniques were used to predict a relatively accurate structure. The Protein Data Bank, a database of protein amino acid sequences [28], was used to retrieve the Human ABCB-1- P-gp protein sequence. The FASTA sequence of human ABCB1-Pgp was retrieved from UniProt (UniProtKB accession no: P08183.3) (https://www.uniprot.org). Next, Blastp alignment was used to find the most closely related protein sequence to ABCB-1. The best similar template of human ABCB-1 P-gp homologue of *S. typhi* ABC-TPA was selected based on the sequence coverage, identity and E-value. Sequence alignment of the Human ABCB1-P-gp protein and *S. typhi* ABC-TPA protein was performed using ClustOmega [29].

*Transmembrane prediction and domain identification*. The predicted amino acid sequence derived from the *S. typhi* ABC-TPA gene was further subjected to transmembrane domain prediction using the TMHMM server. This step aimed to identify potential transmembrane regions within the protein structure, crucial for understanding its topology and functional architecture. The InterProScan tool was also employed to predict protein families and domains in the selected amino acid sequence. This comprehensive analysis helped identify and annotate specific conserved protein domains, enriching the understanding of the protein’s functional context and potential structural motifs before finalising the template for subsequent modelling processes.

*Structure prediction*. Given the unavailability of a resolved crystal structure for *S. typhi* ABC-TPA, a local implementation of AlphaFold was deemed necessary for precise structure prediction. The amino acid sequence specific to *S. typhi* ABC-TPA was formatted and processed for local deployment of the AlphaFold software. This involved downloading and configuring the AlphaFold software locally within our computational environment. Utilising the pre-trained models and algorithms incorporated within AlphaFold, the protein structure prediction was executed using default parameters. This local approach provided an in-depth analysis, ensuring a thorough and detailed structural prediction of *S. typhi* ABC-TPA [30].

*Model validation*. The quality of the modelled structures of TPA was evaluated using SAVES v6.0, a Meta server that runs six applications for examining and validating protein structures. The applications used by SAVES v6.0) (accessed on January 15, 2021, at https://servicesn.mbi.ucla.edu/SAVES/), the following tools were used to assess the quality of the structure ERRAT [31], PROVE [32], PROCHECK [33] and VERIFY 3D [34]. The model with the highest overall scores for its quality parameters was selected to represent *S. typhi* ABC-TPA because it was the most reasonable model for downstream analysis.

*Superimposition*. Even though sequence similarity between the template and our protein was low (about 27%), they both had a structural similarity. The structural comparison of the *S. typhi* ABC –TPA model was done in FATCAT online server (http://fatcat.godziklab.org/2022) by superimposing the *S. typhi* ABC-TPA models over the crystal structure of the human ABCB1 [35].

*Molecular dynamic simulation*. To obtain the structurally stable conformation of Alpha fold-predicted *S. typhi* TPA protein was submitted to molecular dynamics simulations. For the molecular dynamics simulations, GROMACS 2019.6 was employed. The AMBER force field ff99SB-(ILDN) was used for the protein preparation. A triclinic box was created around the protein, with a minimum distance of 1.0 nm between protein atoms and grid box boundaries. Box was filled with 3-point water (TIP3P) and Cl- ions to produce a neutralized aqueous environment. The system’s energy was neutralized using the steepest descent minimization algorithm. The system was energy minimized using the Steepest Descent Algorithm with a 10.0 kJ/mol cut-off energy value for a maximum of 500 ps. After minimization, 100 ps NVT and NPT equilibration, each with position restraint was applied for protein. Subsequent MD simulation of the protein was performed for 50 ns of constant pressure equilibration without constraints to relax the system using the leap-frog integrator algorithm. The root mean square fluctuations (RMSF), root mean square deviation (RMSD), and the radius of gyration (Rg) was calculated between the protein and ligands using GROMACS [36, 38].

### Docking analysis

#### Ligand preparation

The structure of three lignans, SA, SB, and SC, considered for the study, were retrieved from PubChem (www.pubchem.com accessed on May 15, 2021) in SDF format and implied as ligands. To perform molecular docking, ligands were converted from SDF to PDB format using Open Babel at pH 7 [36].

#### Protein preparation

The stable three-dimensional experimental crystal structure of *S. typhi* ABC-TPA was prepared for docking. The discovery studio was used to prepare and convert the protein structure, and polar hydrogens were added to the protein structure by removing water molecules.

#### Binding sites prediction and molecular docking

The CB Dock server (http://clab.labshare.cn/cb-dock/php/index.php; accessed February 21, 2022) uses a novel curvature-based cavity detection approach to predict the binding sites of a given protein and calculate the centers and sizes [36]. The server integrates with AutoDock Vina and has been exactly adjusted to achieve a model success rate of more than 70%. CB-Dock is a cavity-based detection molecular docking technique that uses the entire protein surface to dock ligands into a receptor binding site. Five potential coupling cavities were found using the PDB format of protein and SDF format of ligand to perform the docking analyses. The protein-ligand interactions were determined using discovery studio with a default parameters: as cutoff length of 3 angstroms Å for hydrogen bond and angle cutoff of 120 degree and a distance of 2.5 to 3.5A for van der Waals interactions which was calculated based on the sum of the van der Waals radii of the interacting atoms ± 0.5Å. After docking, the best conformation was selected based on the lowest Vina score obtained. The protein and ligands were then visualized [37]. The binding strength of the ligand was calculated as a negative score (kcal/mol).

### Molecular dynamic simulations of the docked complex

The stability of *S. typhi* ABC-TPA was investigated using a molecular dynamics simulation for 100 ns, with the three ligand-protein complexes having the lowest binding energies. The molecular dynamics simulations were performed using GROMACS 2019.6. Amber force field ff99SB-(ILDN) and Antechamber were used for the protein and ligand preparation, respectively. The ligand parameters were generated by ACPYPE (AnteChamber Python Parser Interface) 2022.3.11 using GAFF2 (General Amber Force Field ver2). A triclinic box was generated around the protein with a minimum of 1.0nm distance between protein atoms and margins of the grid box. In the triclinic box, ligands and protein were combined and centralized. Box was filled with 3-point water (TIP3P) and Cl- ions to produce a neutralized aqueous environment. The system’s energy was balanced using the steepest descent minimization algorithm. The system was energy minimized using the Steepest Descent Algorithm with a 10.0 kJ/mol cut-off energy value for a maximum of 500 ps. Complexes were then run through NVT and NPT equilibration for 100 ps each with position restraints applied for both protein and ligand. Finally, the MD Simulation run of 100 ns was performed for each selected protein-ligand complex using the leap-frog integrator algorithm. Using GROMACS, RMSF, RMSD, and the Rg between the protein and ligands were calculated [38].

### ADMET screening and toxicity prediction

The drug-likeness is guided by molecular properties. All tested compounds SA, SB and SC do not violate Lipinski rule as their values fall in the normal range. Furthermore, these compounds are in the optimal range of the physiochemical space thus, these compounds could be considered as lead compounds. ADMET properties were predicted using ADMET lab 2.0. In terms of absorption all the compounds SA, SB and SC have greater permeability while the substances SA and SB will have higher HIA scores than SC. In term of PPB and VD, the compounds SC and SB tightly bound to protein and blood components as compared to SC while the least BBB values for SA and SB as compared to SC [39].

## Results

### Predictive modelling and validation of *S. typhi* ABC-TPA protein structure

**Fig 1:**
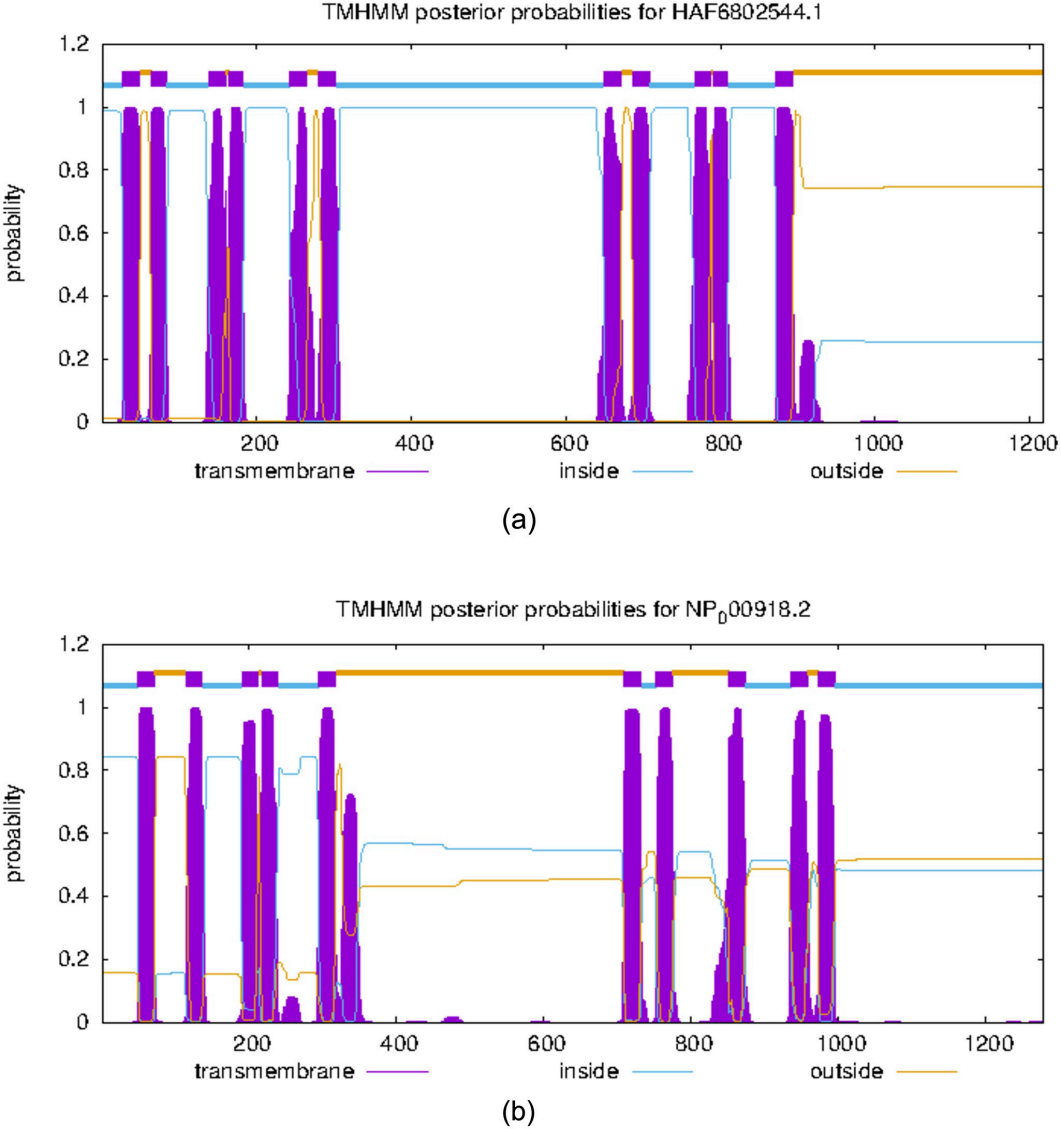
(a) Transmembrane locations of TPA-ABC of *S. typhi* (b) Transmembrane locations of P-gp.

Despite the low sequence identities obtained from the BLAST search, identifying identical structures for modelling the *S.typhi* ABC-TPA protein was proved to be challenging [40]. The Blast score with the highest E-values showed less than 30% sequence identity with the P-gp Table 1. Consequently, we strategically leveraged structural similarities, such as transmembrane regions and the N-terminus, to select the most appropriate template, with the sequence ID of HAF6802544.1. It is listed as TPA in NCBI gene bank with an amino acid length of 1218. Similar to P-gp it has 6 transmembrane domains with three of them evenly spaced in the start and inner ATP binding region and the three spaced out afterwards following an inner ATP binding region as can be seen in Fig 1. Fig 1(a) and 1(b) shows the transmembrane domain locations of ABC to TPA of *S. typhi* and P-gp respectively.

**Table 1:** BLAST results showing best selected template of P-gp homologue of *S. typhi* TPA. The best template is selected based on the E-value, sequence identity, and BLAST score.

Sequence ID	Protein	E-values	Blast score	Identity
WP001111803	*Salmochelin/enterobactin* export ABC transporter IroC (*Salmonella enterica*)	1e-108	372 1	20.8%
HAF6802544.1	TPA: ABC transporter protein (*Salmonella* enterica serovar *typhi*)	8e-108	370	27.98%
EBL5772985.1	ABC transporter ATP-binding protein (*Salmonella* enterica serovar *typhi*)	1e-107	369	28.42%
HAF4997026.1	TPA: ABC transporter *Salmonella* enterica serovar *typhi*)	4e-107	368	28.04%
ECN6611564.1	ABC transporter ATP-binding protein (*Salmonella* enterica serovar *typhi*)	4e-107	368	28.04%
ECD7319002.1	ABC transporter ATP-binding protein (*Salmonella* enterica serovar *typhi*)	5e-107	368	28.04%
HAF3941128.1	TPA: ABC transporter ATP-binding protein *Salmonella* enterica serovar *typhi*)	5e-107	368	27.77%
WP_0_01111816.1	*Salmochelin/enterobactin* export ABC transporter IroC (*Salmonella*)	5e-107	368	27.77%
WP_0_24154731.1	*Salmochelin/enterobactin* export ABC transporter IroC (*Salmonella* enterica)	1e-106	367	27.74%
HAA1104969.1	TPA: ATP-binding cassette domain-containing protein (*Salmonella* enterica serovar *typhi*)	9e-107	367	28.08%

**Fig 2:**
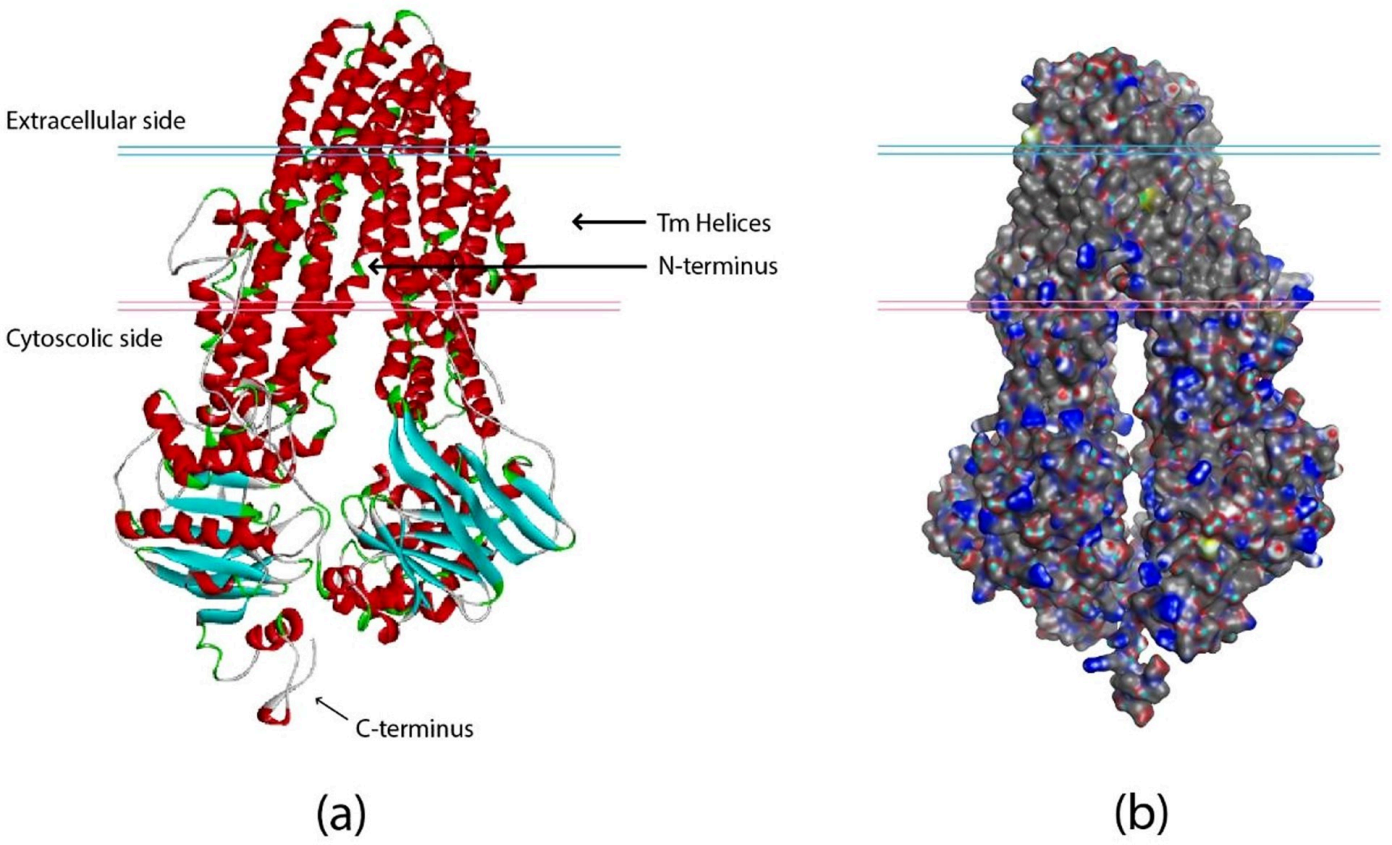
(a) Cartoon view of the predicted tertiary structure of the ABC-TPA from the technique used, Alpha Fold; respectively and (b) Surface representation.

Using InterProScan (https://www.ebi.ac.uk/interpro/), protein family domains were aligned and tested, and TPA-ABC (HAF6802544) was classified as a type 1 protein exporter. The sequence similarity was low due to the underlying structural similarity of ABC-TPA to P-gp. Using this selected sequence, We then employed AlphaFold, a State-of-the-Art Machine learning-based tool, to predict the three-dimensional structure of the previously unreported *S. typhi* ABC-TPA. The resulting structure depicted through alphafold model shown in Fig 2, underwent meticulous validation through SAVES v6.0 utilizing diverse structural assessment tools including ERRAT, VERIFY 3D, PROVE and PROCHECK.

**Fig 3:**
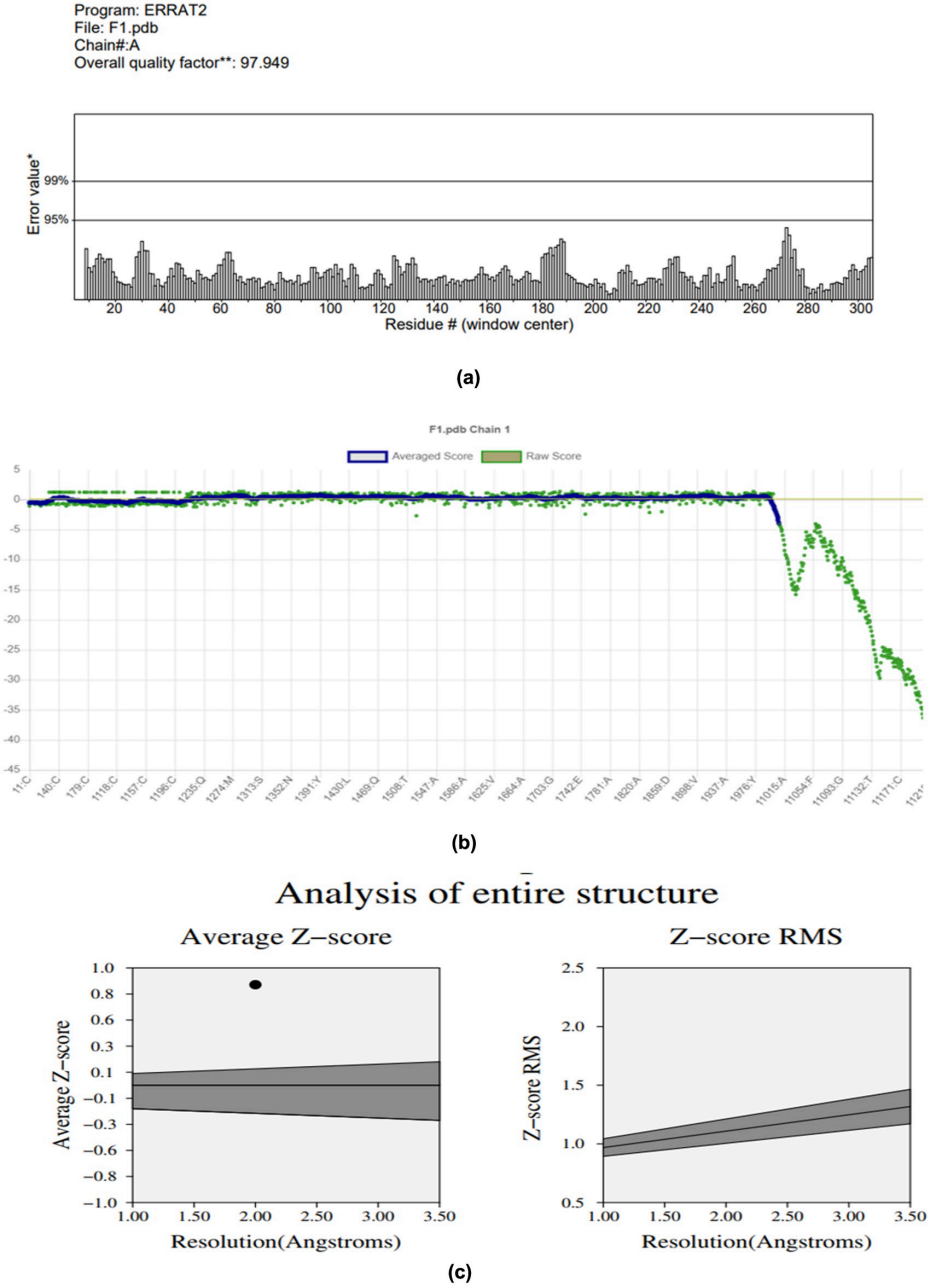
ERRAT plot of the overall quality factor of the modeled structure of ABC-TPA is shown. (a) The graph represents the error values of model residues predicted by ERRAT. The “x” axis indicates the amino acid sequences, while the “y” axis presents the error values. (b) The graph represents the Verify D%. (c) The graph represents the Prove scores.

SAVES v6.0 uses different tools(such as ERRAT, VERIFY 3D and PROVE to assess the quality of structural validation of proteins including quality, potential error, and accuracy. ERRAT was applied for realizing non bonded interactions between several atom types (CC, CN, CO, NN, NO, and OO). ERRAT with an overall score of 97.949% Fig 3(a) was obtained and reported as a percentage of the protein for which the estimated error value is less than the 95% rejection limit. VERIFY 3D program was used to analyze the compatibility of an assembled atomic model (3D) with its corresponding amino acid primary sequence (1D). It assessed the residue packing, atomic interactions, and solvent accessibility to analyze the overall quality and provide structural reliability. (VERIFY 3D scores of 80% obtained showed that the residues have an average 3D-1D score ≥0.0 Fig 3(b). PROVE analysis computes the volumes of atoms in macromolecules using an algorithm that treats the atoms as hard spheres and calculates a statistical Z-score deviation of the model from a highly resolved and refined PDB-deposited structure. PROVE x score was found to be 4.9. Fig 3(c).

The structural integrity of the AlphaFold-predicted ABC-TPA model was comprehensively evaluated. ERRAT analysis demonstrated an outstanding overall quality score of 97.949% Fig 3(a), affirming the model’s reliability in representing non-bonded interactions between various atom types.

Further validation through VERIFY 3D indicated substantial compatibility between the atomic model and the primary amino acid sequence, with 80% of residues exhibiting averaged 3D-1D scores ≥ 0.0 Fig 3(b). Additionally, the PROVE analysis rendered a favorable Z-score of 4.9, bolstering confidence in the model’s structural accuracy Fig 3(c). It also analyzes the environment of individual residue and checks the consistency of the local environment with known protein structure.

**Fig 4:**
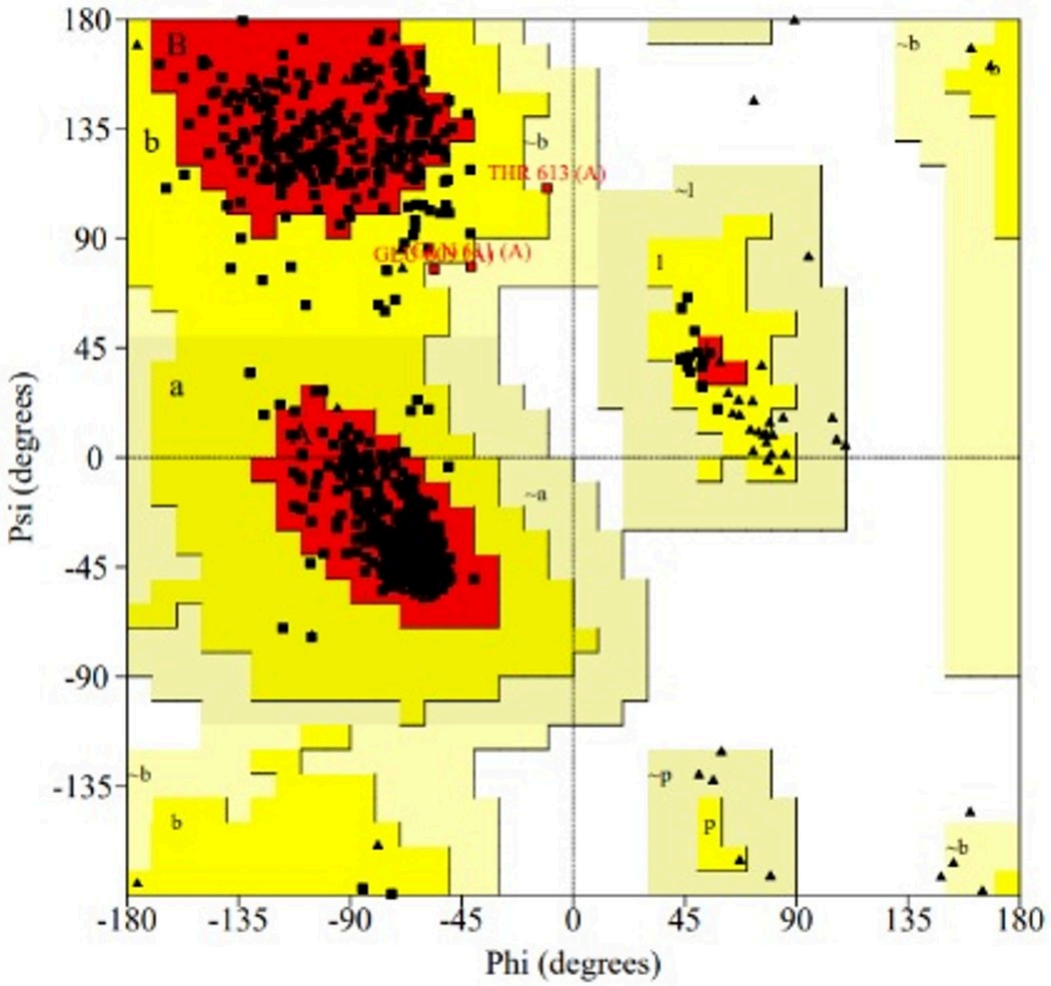
Ramachandran plot of the selected ABC-TPA structure obtained via PROCHECK. The percentages of residues in the most favored regions, additionally allowed regions, generously allowed regions and disallowed regions are 94.4%, 4.9%, 0.3%, and 0.0% respectively.

PROCHECK, on the other hand, provides information on the entire structural geometry by generating Ramachandran plots. These plots are used to phi and psi bond angles to see if the two opposing amino acids are not placed together. PROCHECK analysis provided detailed insights into a favourable structural geometry, showcasing 94.4% of residues in the most favoured regions, further solidifying the favourable stereochemistry of our model as shown in Fig 4 and Table 2.

**Table 2:** Ramachandran plot statistics for the model from the modelling technique. For a model, the number of end residues (excluding Gly and Pro) = 2, Glycine residues = (97), Proline residues = (37), and the total number of residues = 129.

	No. of residues	Percentage
Most favored regions (A, B, L)	1032	94.4%
Allowed regions (a, b, l, p)	53	27.98%
Generously allowed regions (a, b, l, p)	3	0.3%
Disallowed regions	0	0.0%

**Fig 5:**
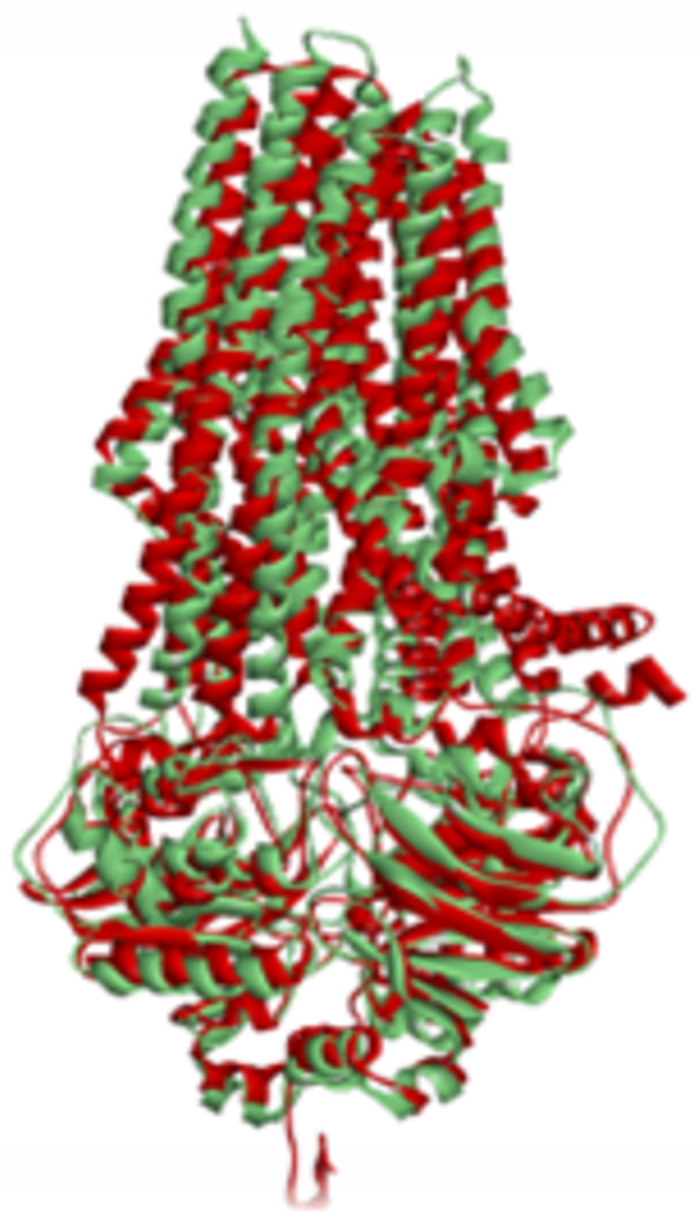
Superimposed protein structures of the *S. typhi* ABC-TPA model generated and the crystal structure of human ABCB1-P-gp. The human P-gp is colored green, and TPA is colored red.

To ascertain structural similarities and potential functional analogies, we superimposed the modelled *S. typhi* ABC-TPA with human ABCB1-P-g using fatcat (https://fatcat.godziklab.org) Remarkably, the initial and optimized RMSD values of 3.43 and 3.23 fell significantly below the accepted threshold of 5.0, reaffirming strong structural alignment shown in Fig 5. This structural resemblance suggests plausible analogous functions, specifically in efflux pump activities aiding *S. typhi* evasion of antibiotics.

Given the absence of X-ray resolution for the ABC-TPA protein and considering the reliance on AlphaFold prediction, our study employed an essential step to assess the structural integrity and stability through Molecular Dynamics (MD) simulations, a significant endeavor in protein structure validation.

The MD simulations, conducted over 50 nanoseconds using GROMACS 5.1, provided invaluable insights into the dynamic behavior of the protein. These simulations, aimed at capturing systems close to their native conformations, involved various structural analyses crucial for evaluating the predicted structure’s robustness.

**Fig 6:**
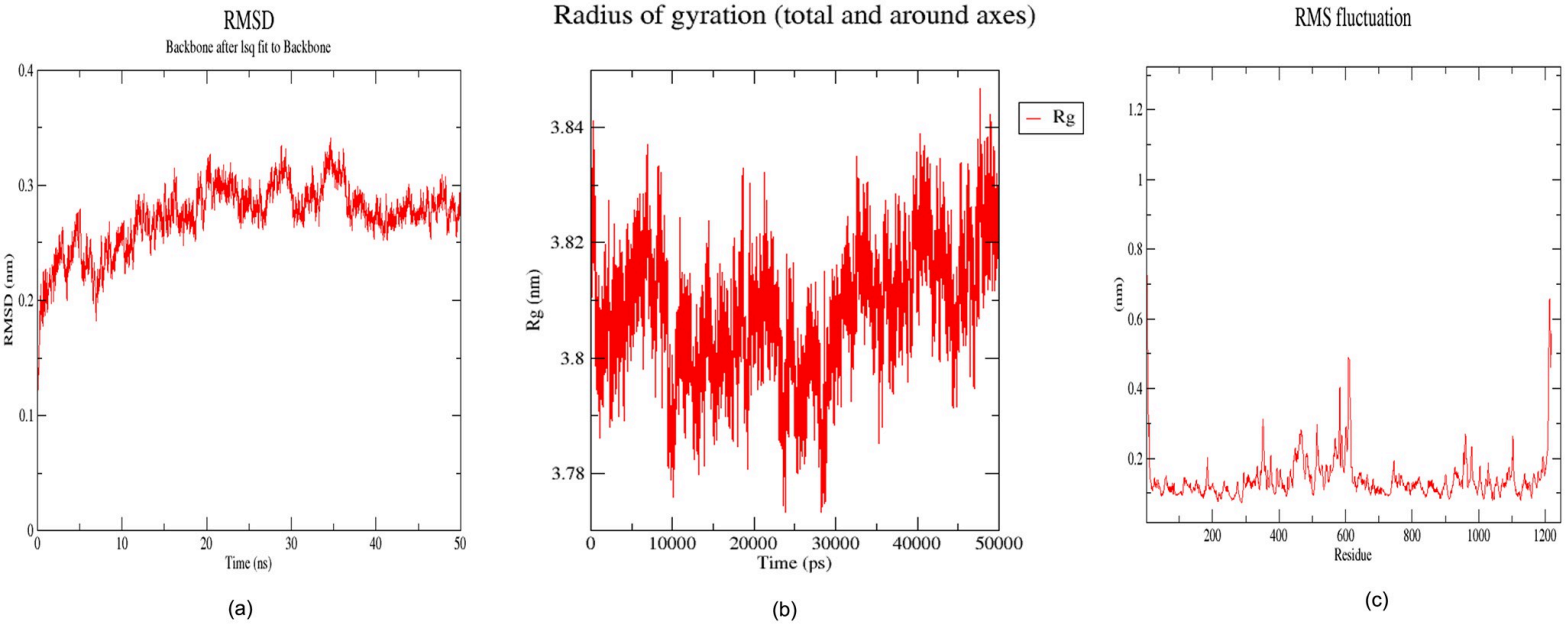
The root mean square deviation (RMSD), radius of gyration (Rg), and root mean square fluctuation (RMSF) graphs of the ABC-TPA generated over a 50 ns molecular dynamics simulation. (a) RMSD versus time graph of ABC-TPA; (b) Radius of gyration (Rg) versus time graph of ABC-TPA; and (c) Analysis of RMSF trajectories of residues of ABC-TPA.

RMSD analysis, a pivotal measure assessing structural deviations from a reference structure over time, demonstrated a consistently low RMSD of 0.28 nm throughout the 50 ns trajectory 100 ps Fig 6(a). This observation signifies the stability and overall structural fidelity of the predicted ABC-TPA protein, reinforcing confidence in the reliability of the AlphaFold-based prediction.

Furthermore, the Rg analysis indicated a consistently maintained Rg range between 3.77 nm and 3.84 nm Fig 6(b), highlighting the protein’s retained compactness and structural stability over the simulation period. The negligible fluctuations observed in Rg underscore the maintained integrity of the folded protein structure.

The RMSF plot revealed localized fluctuations in specific regions of the ABC-TPA protein Fig 6(c). Notably, residues within indexes 352, 515, 584, 611, and 610 displayed varying degrees of flexibility, while the region spanning residues 448–456 exhibited the highest fluctuations, suggesting their potential involvement in ligand binding interactions.

### Schisandrin A, B, and C as potential inhibitors for *S. typhi* ABC-TPA

Building upon the structural validation and modelling of the *S. typhi* ABC-TPA protein, the investigation extends to explore Schisandrin A, B, and C as potential inhibitors for this efflux pump homologue. These lignans, sourced from botanical origins such as Schisandra chinensis, boast diverse biological activities and are speculated to hold therapeutic significance. Notably, within the realm of antibacterial resistance, their role as Efflux Pump Inhibitors (EPIs) has garnered attention, suggesting the potential disruption of efflux pump-mediated antibiotic resistance mechanisms in bacterial pathogens. The selection of these lignans for investigation stems from their known bioactivities and the anticipated capacity to modulate efflux pumps, presenting a promising avenue in combating antibiotic resistance. However, before probing their interactions with efflux pump homologues, a comprehensive understanding of their drug-likeness and essential properties is imperative.

Table 3 depicts the drug likeness properties of the compounds (SA, SB and SC) using a free web ADMET lab 2.0 server. It can be seen all the compounds are within Lipinski rule’s typical range. The Lipinski rule of 5 indicates that poor absorption or penetration is more likely when a molecule has more than 5 H-bond donors (NH and OH), MW is over 500, Log P is over 5, and more than 10 H-bond acceptors (the sum of N and O). In medicinal chemistry, the pan assay interference compounds (PAINS) structural alerts have been utilized to anticipate the presence of unstable, reactive, and hazardous fragments. PAINS descriptors show zero warnings for all compounds SA, SB, and SC, proposing additional encouraging signs that they could be therapeutic candidate.

**Table 3:** Physicochemical, pharmacokinetics, and medicinal chemistry properties of the compounds SA, SB and SC using ADMET lab 2.0 server.

Compounds	Mw g/mol	HBA	HBD	TPSA	lOgP	mr	Lipsinki	PAINS	NP score	Golden Triangle
SA	416.22	6	0	55.38	4.436	16	Yes	0	0.795	Yes
SB	00.19	6	0	55.38	4.595	19	Yes	0	1.242	Yes
SC	384.16	6	0	55.38	4.932	32	Yes	0	1.115	Yes

### ADMET prediction

ADMET predictions using the ADMET lab 2.0 server revealed crucial insights into the properties of SA, SB, and SC, and the results are documented in Table 4. ADMET scores indicate potential toxicity of Schisandrins before undergoing in vitro/in vivo analysis, several crucial ADMET parameters were evaluated. Among these, absorption studies, measured through Caco-2 permeability assays, revealed higher absorption rates for SA and SB than SC. Moreover, distribution analysis indicated a wider distribution profile for SC, suggesting its potential for broader systemic presence. Considering metabolism, all compounds share common enzymatic pathways involving various CYP enzymes, cautioning against concurrent administration with lower affinity medications. Importantly, SA exhibited a longer half-life than SB and SC, indicating its potentially sustained presence in the body. These findings collectively suggest that SA and SB exhibit favourable absorption and moderate distribution profiles while SC shows broader distribution potential.

**Table 4:** ADMET properties of the compounds SA, SB, and SC using ADMET lab 2.0 server.

C	Absorption					Distribution		Metabolism					T1/2 h	Excret. MI/min/kg
	Caco 2p (Cm/s)	Pgp (I)	Pgp (s)	HIA	Caco 2p (Cm/s)	BBB	VD (L/KG)	CYP IA2(I) and (S)	2C19(I), (S)	2c9 (I), (S)	2D6	3A4		
SA	-4.783	0.989	0.004	0.006	69.64	0.054	0.684	0.077, 0.988	0.416, 0.948	0.125, 0.888	0.001, 0.925	0.666, 0.91	0.01	5.617
SB	-4.818	0.983	0.001	0.002	80.93	0.057	0.921	0.216, 0975	0.942, 0.912	0.324, 0.926	0.025 0.924	0.918, 0.775	0.08	9.996
SC	-4.876	0.722	0.0	0.001	92.98	0.064	2.247	0.647, 0489	0.997, 0818	0.706, 0.933	0.324, 0.917	0.951, 0.501	0.08	13.62

### Molecular docking analysis of Schisandrin A, B, and C with *S. typhi* ABC-TPA

**Fig 7:**
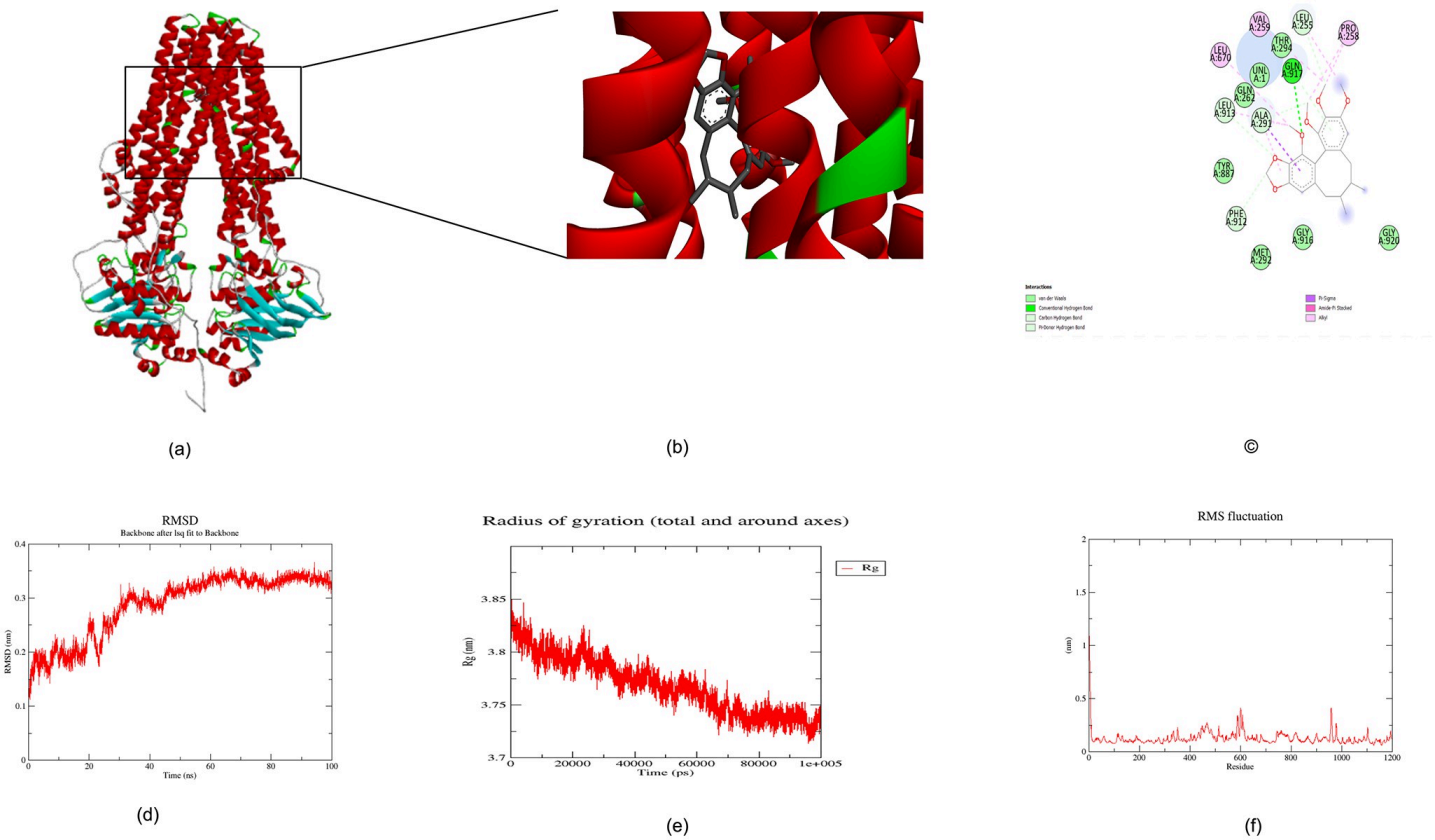
Molecular docking between *S. typhi* ABC-TPA and SA, (a) 3D structure between ABC-TPA and SA, (b) cavity displaying TPA and SA’s bonding (c) Protein–ligand interactions using Discovery studio server. Protein–ligand interactions of TPA-SA is represented, highlighting the key residues involved in the interaction, (d) The root mean square deviation (RMSD), (e) radius of gyration (Rg), (f) and root mean square fluctuation (RMSF) graphs of the TPA-SA complex generated over a 100 ns molecular dynamics simulation.

**Fig 8:**
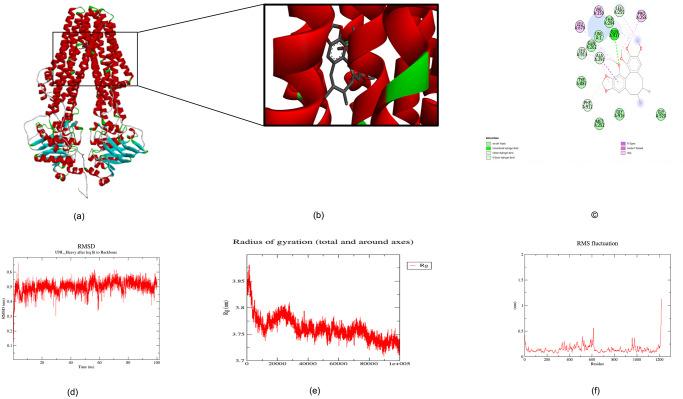
Molecular docking between *S.typhi* TPA-SB, (a) 3D structure between ABC-TPA and SB, (a) cavity displaying TPA and SB’s bonding (c) Protein–ligand interactions using Discovery studio server. Protein–ligand interactions of TPA-SA is represented, highlighting the key residues involved in the interaction, (d) The root mean square deviation (RMSD), (e) radius of gyration (Rg), (f) root mean square fluctuation (RMSF) graphs of the TPA-SB complex generated over a 100 ns molecular dynamics simulation.

**Fig 9:**
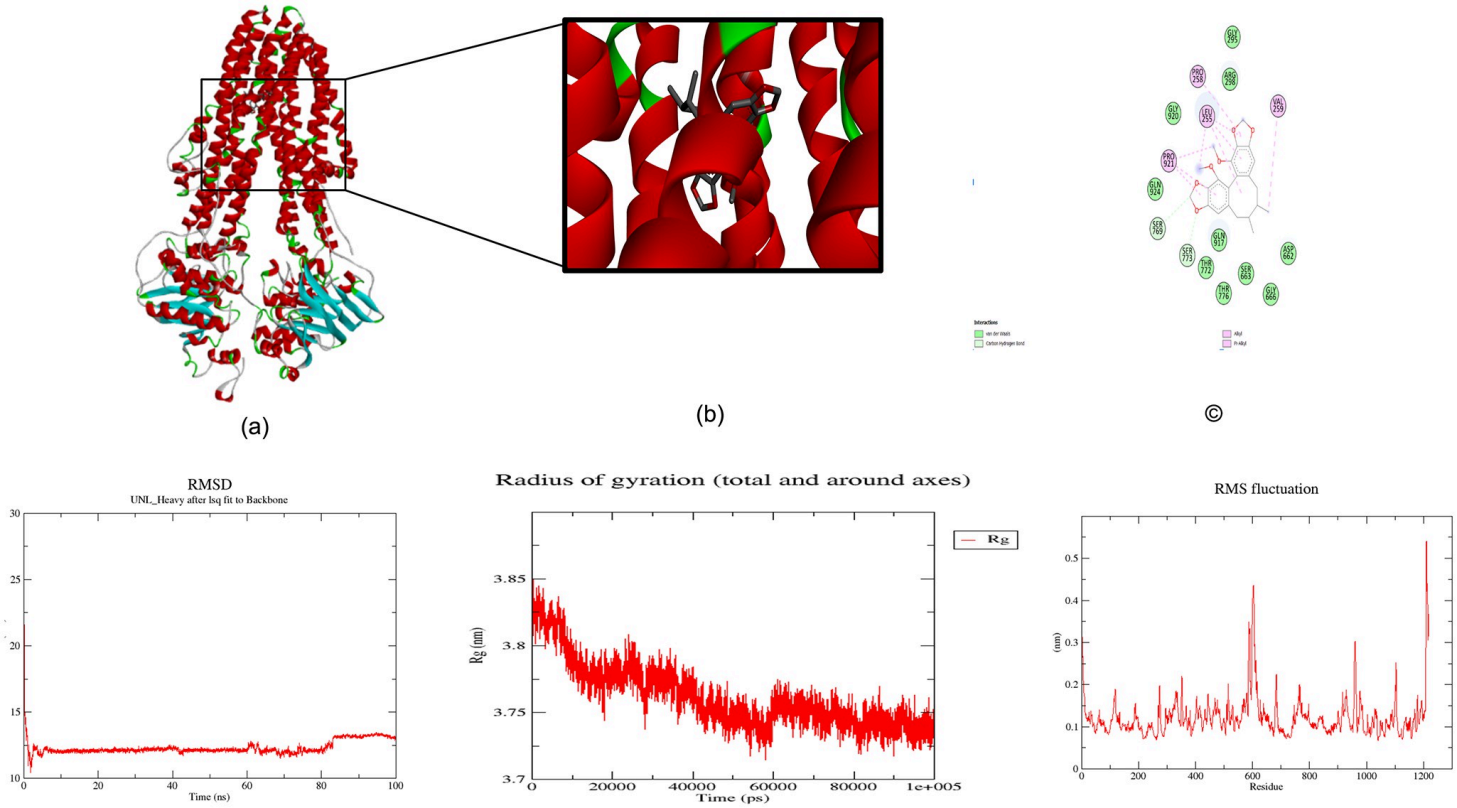
Molecular docking between *S.typhi* TPA-SC, (a) 3D structure between ABC-TPA and SC, (b) cavity displaying TPA and SC’s bonding (c) Protein–ligand interactions using Discovery studio server. Protein–ligand interactions of TPA-SC is represented, highlighting the key residues involved in the interaction, (d) The root mean square deviation (RMSD), (e) radius of gyration (Rg), (f) and root mean square fluctuation (RMSF) graphs of the TPA-SC complex generated over a 100 ns molecular dynamics simulation.

Following the comprehensive assessment of the drugs’ ADMET profiles and drug-likeness, the investigation progressed towards exploring potential interactions of SA, SB, and SC with the human P-gp homologue of ABC-TPA. This exploration was built upon the earlier structural prediction and thorough assessment of the *S. typhi* ABC-TPA protein. Employing the CBDOCK online server, molecular docking studies were conducted to unravel potential binding interactions. Figs 7(a), 8(a) and 9(a) illustrate the outcomes of the CB-DOCK analysis, shedding light on the putative binding modes of these lignans with the efflux pump homologue.

Five possible interaction models based on protein-ligand binding affinity prediction using a curvature-dependent surface-area model had been made between ABC-TPA (protein) and SA, SB, and SC (as ligands). The values of Binding energy were obtained as (Vina’s score). The docked complexes with the least binding energy were -7.l kcal/mol and -7.4 kcal/mol -8.2 kcal/mol, respectively, for SA, SB and SC. The lower the value, the stronger the binding effect; hence more negative values are more favorable and represent stronger interactions. Interactions were furthermore visualized using discovery studio and can be seen in Figs 7(c), 8(c) and 9(c). Moreover the most significant active cavity were selected respectively Table 5.

**Table 5:** The lowest binding energy obtained as Vina scores and cavity size obtained from the web interface of CB-DOCK.

Complex size	Vina Score kcal/mol	Cavity
TPA-SA	-7.1	13696
TPA-SB	-7.4	5956
TPA-SC	-8.2	13696

Table 6 depicted drug toxicity which is a significant contributor to drug attrition during the discovery and development phases. By using the ADMET lab 2.0 server, the toxicity risk of the compound SA, SB, and SC were quantitatively evaluated. The findings of the hERG-based toxicity assessment demonstrate that SA, SB, and SC were the least dangerous and have no cardiotoxicity. Ames is an assay to determine a substance’s mutagenicity, or its capacity to cause genetic damage and mutations. The compound SA, SB, and SC do not have any mutagenic properties. The median lethal dose (LD50), which represents acute toxicity, is the dose of a tested substance that instantly kills 50% of the treated animals. Compared to SB and SC, which had LD50 values of 5.147 mg/L and 5.35 mg/L, respectively, SA has the highest (LD50) value of 5.8 mg/L.

**Table 6:** Toxicity prediction of compounds SA, SB and SC using ADMETlab server 2.0.

Compounds	Toxicity			
	hERG	HH-T	AMES	LD50mg/L
SA	0.37	0.47	0.88	5.801
SB	0.25	0,081	0.131	5.147
SC	0.136	0.147	0.247	5.35

### Molecular dynamic simulation of docked complexes

#### TPA-SA complex

TPA docked with SA with the binding energy of −7.1 kcal/mol. The binding of SA to the TPA efflux pump is characterised by a series of interactions with specific amino acids. Hydrogen bonds were observed with GLN917 and ARG298, which are crucial for the stability of the binding. Carbon-hydrogen bonds were formed with THR776, SER663, LEU255, and GLN920, while van der Waals forces involved GLN294, THR772, GLY666, ASP662, ALA256, ALA291, and GLY295. Additionally, alkyl interactions were noted with PRO258, PRO921, and VAL259, contributing to the overall affinity of SA for the efflux pump. These interactions collectively facilitate the compound’s potential inhibitory action on the pump’s activity Fig 7(c), Table 7.

**Table 7:** The binding energies and intermolecular bonds between selected compounds and ABC-TPA.

Compounds	Binding Energy	Interaction Residues	Interaction Types
SA	-7.1	GLN294, THR776, SER663, LEU255, GLY920	Conventional Hydrogen bond Carbon Hydrogen bond Vander Walls Alkyl
		GLN294, THR776, SER663, LEU255, GLY920	
		GLN294, THR776, SER663, LEU255, GLY920	
		PRO258, PRO921, VAL259	
SB	-7.4	GLN917	Conventional Hydrogen Bond Carbon Hydrogen bond Vander walls Alkyl
		GLN294, THR776, SER663, LEU255, GLY920	
		GLN294, THR776, SER663, LEU255, GLY920	
		LEU670, VAL259, PRP258	
SC	-8.2	SER769, SER773, GLY295, GLY920, GLY666, GLN917, GLN924, THR772, THR776, ARG298, SER663, ASP662	Carbon Hydrogen bond Alkyl
		AD	

The MD simulation was run for 100 ns for all the complexes to see how well bound the ligands were with the protein; we assessed them on three parameters, RMSD, Rg and RMSF. For the TPA-SA complex, the average RMSD was 0.32 nm with little fluctuation until the end of the100 ns simulation period Fig 7(d). The Rg only increases by 3.73 nm, about 100 ns. In addition, there was no significant change in the radius of the gyration TPA-SA complex (Fig 6e). However, the RMSF plot for the SA complex revealed some fluctuations were observed in amino acid regions (588, 601, 609, and 959) of the TPA-SA complex Fig 7(f). The highest fluctuation were observed between residues (435–482). As can be seen, most of the fluctuations were small, less than 0.5 nm around the binding site, and the ligand remained within the protein after the MD simulation. This means that the docked ligand is firmly bound and can act as a suitable inhibitor of the protein. For more information, see S1 File.

### TPA-SB complex

TPA docked with SB with the binding energy of −7.4 kcal/mol. In this graphical representation Fig 8(c), Table 4 depicted that SB was bound to the TPA efflux pump, forming hydrogen bonds predominantly with GLY666 and SER663. It also establishes carbon-hydrogen bonds with ASP662, contributing to the molecule’s stability within the binding site. Van der Waals forces involved interactions with amino acids like VAL700 and ALA665, while pi-anion interactions are seen with GLN917. These interactions are crucial for the positioning and potential inhibitory action of SB on the efflux pump, with each type of bond playing a role in the compound’s binding affinity and specificity. For the TPA-SB complex, the average RMSD was 0.34–0.6 nm with fluctuations until the end of the100 ns simulation period Fig 8(d). The Rg values of the TPA-SB complex experienced a decline from the start and, maintained a steady Rg of an average of 3.71 nm until about 100 ns. There was a significant change in radius of gyration TPA-SB complex Fig 8(e). The RMSF plot for SB complex revealed that some degree of fluctuations were observed in regions of the TPA-SB complex Fig 8(f). Fluctuations were observed at regions from residue index (514, 614, 958, 959, 978, 979 and 980). As can be seen, most of the fluctuations were small and the ligand remained within the protein after the MD simulation. This means that the docked ligand is firmly bound and can act as a suitable inhibitor of the protein. For more information, see S1 File.

#### TPA-SC complex

TPA docked with SC with the binding energy of −8.2 kcal/mol. The image Fig 9(c), Table 7 shows SC engaged with the TPA efflux pump through a network of molecular interactions. Carbon-hydrogen bonds are present with SER769 and SER773, while alkyl interactions are formed with PRO258, PRO921, and LEU255. Additionally, pi-alkyl interactions are seen with VAL259, indicating a close contact with aromatic components. This pattern of interactions helps to secure SC within the binding site of the efflux pump, which could be significant for its function or inhibition. The RMSD values of SC complex was observed to rise 21.5 nm at the start and experienced a fall with RMSD of 12.5 nm until about 60 ns, and after 60 ns some fluctuations and structural changes were observed until at the end of 100 ns simulation period with average RMSD of 13.4nm Fig 9(d). The Rg values of the TPA-SC complex experienced a decline from 3.85 nm to about 3.75 nm until about 100 ns. There was a significant change in radius of gyration TPA-SC complex Fig 9(e). The RMSF plot for SC complex revealed that some degree of fluctuations were observed in regions of the TPA-SC complex Fig 9(f). Fluctuations were observed at regions from residue index 599 and 961. As can be seen, most of the fluctuations are large and the ligand remains within the protein after the MD simulation. This means that the docked ligand cannot act as a suitable inhibitor of the protein.

The MD simulations of the ABC-TPA protein bound to three different ligand complexes, namely SA, SB, and SC, which provide valuable comprehensions into their binding manners and stability. The average RMSD values of complexes ranging from 0.32 nm to 13.4 nm. TPA-SA and TPA-SB complexes exhibited relatively stable and low RMSD and most favorable binding characteristics with insignificant changes in Rg and localized fluctuation in specific regions. These results suggested that ABC-TPA firmly bound to SA and SB, act as potential and suitable inhibitors. While TPA-SC complex exhibits structural changes, and unstable binding interaction with larger fluctuations, and a major alteration in Rg, these findings suggested that SC may not act as an efficient inhibitor for the ABC-TPA. For more information, see S1 File.

## Discussion

Typhoid fever is caused by the Gram-negative bacteria *Salmonella* enterica serovar *typhi*, which contributes significantly to the global health burden [41]. Typhoid fever poses a hazard to public health because to its high rates of morbidity and mortality, particularly in developing countries [42]. There are still many antimicrobials that can effectively treat typhoid fever. However, *S. typhi* that are resistant to empirical antibiotics hinder effective treatment [43]. Identifying and characterising transporters within bacterial systems, particularly in the context of efflux pump mechanisms, are vital steps toward comprehending antimicrobial resistance [44–46]. In our study, the identification of a P-glycoprotein (P-gp) homologue termed ABC-TPA within *S. typhi* was a foundational step. Given the absence of experimentally resolved 3D structures for ABC-TPA, we employed a combination of bioinformatics tools and modelling techniques to predict and validate its structure. Initially, exhaustive similarity searches facilitated the identification of a suitable template for homology modelling. The sequence alignment analysis underscored critical parameters sequence coverage, identity, and E-values ultimately leading to the selection of an appropriate template. This chosen template, sharing fundamental structural attributes and sequence homology with ABC-TPA, reinforced the reliability of our modelling strategy. The predicted ABC-TPA structure underwent numerous validations, notably through ERRAT analysis, endorsing our model’s overall quality and reliability [47]. Molecular dynamics (MD) simulations further assessed the modelled ABC-TPA’s structural stability and conformational dynamics, affirming its structural robustness and suitability for subsequent analyses.

The successful identification, homology modelling, and rigorous validation of the ABC-TPA structure established a robust foundation for our subsequent investigations into efflux pump inhibition using lignans. To assess the use of these lignans we tested them comprehensively for drug-likeness assessment and pharmacokinetic profiling through ADMET screening. The evaluation revealed LD50 values of 5.8—5.147 mg/L for all three lignans. Notably, Schisandrin A (SA) exhibited a slightly elevated margin of safety and lower acute toxicity compared to Schisandrin B (SB) and Schisandrin C (SC), suggesting promising and favourable toxicity profiles for potential therapeutic use.

Subsequently, molecular docking studies were conducted to find the binding affinity and potential inhibitory interactions of SA, SB, and SC with ABC-TPA. The docking analyses showed favourable binding energies: SA at -7.1 kcal/mol, SB at -7.4 kcal/mol, and SC at -8.2 kcal/mol. Though docking studies give an incomplete idea of how the complex and the protein will act in a living environment, we conducted MD simulation studies to assess. The simulations unveiled crucial insights; the TPA-SA and TPA-SB complexes demonstrated stable interactions, minimal root mean square deviation (RMSD), and consistent gyration (Rg) radius, affirming their potential as effective inhibitors by maintaining stable binding characteristics. Conversely, the TPA-SC complex displayed marked structural alterations, substantial fluctuations, and significant deviations in Rg, indicating a less stable interaction profile and suggesting its potential limitations as an efficient inhibitor [48, 49].

This comprehensive evaluation highlights the encouraging candidacy of SA, and SB, as potential therapeutic agents against the challenging MDR *S. typhi* strains. These findings substantiate the importance of further in-depth investigations to validate their efficacy and suitability in combatting multi-drug resistance mechanisms in *S. typhi*

## Conclusion

In summary, this study explored the efficacy of lignans—Schisandrin A, B, and C—as potential efflux pump inhibitors (EPIs) for the P-gp homologue in *Salmonella* enterica *serovar typhi*. These lignans act as added advantage when given with other drugs. The results from molecular docking and molecular dynamics simulations suggested that Schisandrin A and B establish stable complexes with the target protein, demonstrating potentially effective binding interactions. Conversely, SC revealed a lack of stability in binding and substantial structural alterations. Additionally, ADMET analysis revealed that SA possesses advantageous pharmacokinetic properties. Collectively, these findings underscore the potential of SA and SB in addressing multidrug-resistant strains of *S. typhi*.

## Supporting information

S1 Graphical abstract (TIF)

S1 File Simulation files of Schisandrin A, Schisandrin B and Schisandrin C. (DOCX)
