# Correction: Computational evaluation of efflux pump homologues and lignans as potent inhibitors against multidrug-resistant *Salmonella typhi*

**DOI:** 10.1371/journal.pone.0320534

**Published:** 2025-03-19

**Authors:** Iqra Shafique, Mehak Rafiq, Nosheen Fatima Rana, Farid Menaa, Fatemah Almalki, Alya Aljuaid, Sulaiman Mohammed Alnasser, Amenah S. Alotaibi, Madahiah Bint E. Masood, Tahreem Tanweer

There is an error in affiliation 5 for author Sulaiman Mohammed Alnasser. The correct affiliation 5 is: Department of Pharmacology and Toxicology, College of Pharmacy, Qassim University, Qassim, Saudi Arabia.

## References

[1] Shafique I, Rafiq M, Rana NF, Menaa F, Almalki F, Aljuaid A, et al. Computational evaluation of efflux pump homologues and lignans as potent inhibitors against multidrug-resistant *Salmonella typhi*. PLoS One. 2024;19(6): e0303285. doi: 10.1371/journal.pone.0303285 38917154 PMC11198855

