# Supplementary figures and images for: Computational evaluation of efflux pump homologues and lignans as potent inhibitors against multidrug-resistant Salmonella typhi
Source: PLoS One. 2024 Jun 25;19(6):e0303285. doi: 10.1371/journal.pone.0303285 (PMC11198855; doi:10.1371/journal.pone.0303285)

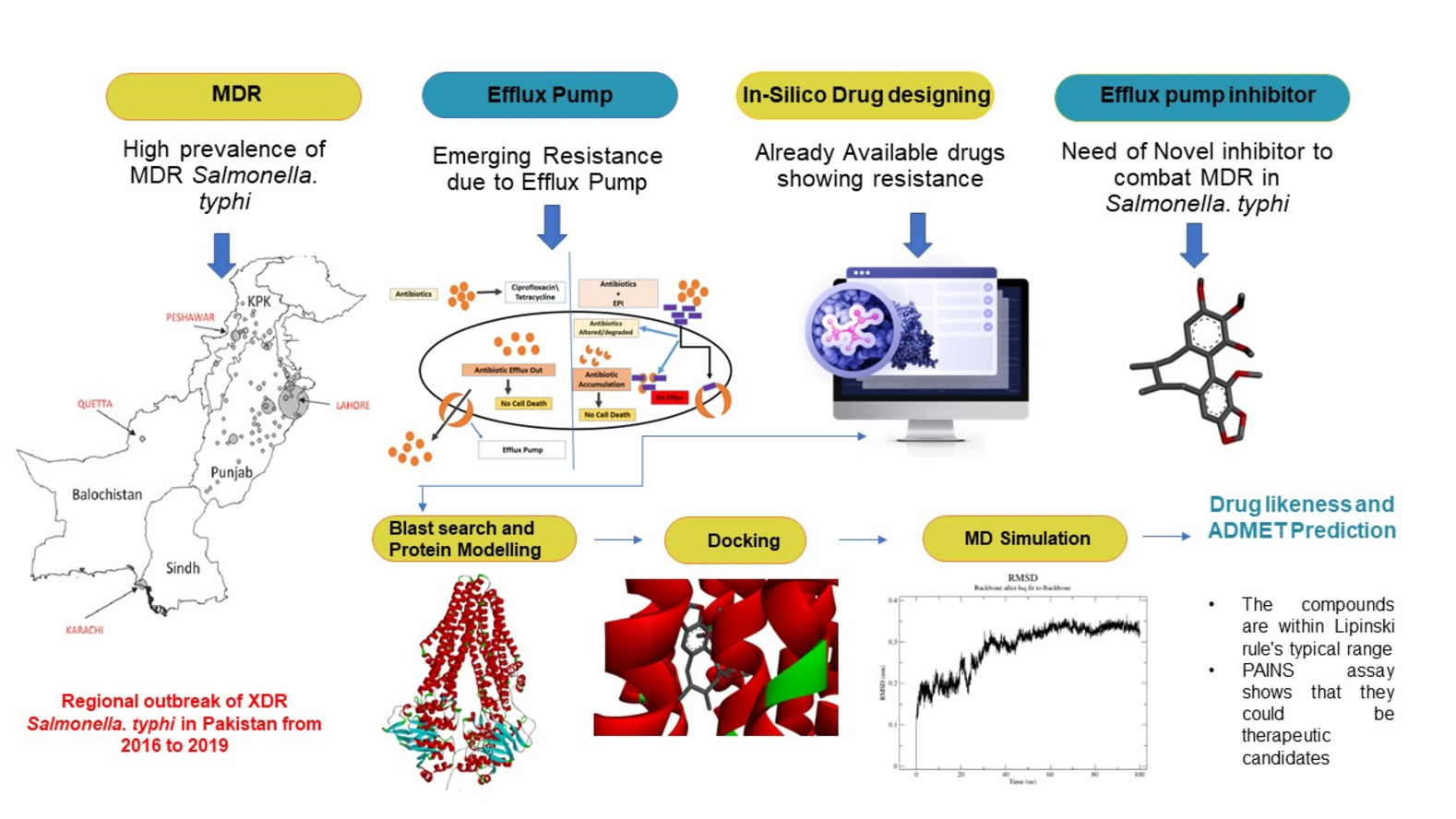

Supplement: S1 Graphical abstract — (TIF) [file pone.0303285.s001.tif]
