# Supplementary material for: Computational evaluation of efflux pump homologues and lignans as potent inhibitors against multidrug-resistant Salmonella typhi
Source: PLoS One. 2024 Jun 25;19(6):e0303285. doi: 10.1371/journal.pone.0303285 (PMC11198855; doi:10.1371/journal.pone.0303285)
Supplement: S1 File — (DOCX) [file pone.0303285.s002.docx]

10.6084/m9.figshare.25243309

<https://figshare.com/s/71fd8270830200df6059>
